# Recurrence dynamics does not depend on the recurrence site

**DOI:** 10.1186/bcr2152

**Published:** 2008-10-09

**Authors:** Romano Demicheli, Elia Biganzoli, Patrizia Boracchi, Marco Greco, Michael W Retsky

**Affiliations:** 1 Department of Medical Oncology, Fondazione Istituto di Ricovero e Cura a Carattere Scientifico Istituto Nazionale Tumori, via Venezian 1, Milano 20133, Italy; 2 Fondazione Istituto di Ricovero e Cura a Carattere Scientifico Istituto Nazionale Tumori, via Venezian 1, Milano 20133, Italy; 3 Medical Statistics and Biometry, Università di Milano, via Venezian 1, Milano 20133, Italy; 4 Breast Surgery, Fondazione Istituto di Ricovero e Cura a Carattere Scientifico Istituto Nazionale Tumori, via Venezian 1, Milano 20133, Italy; 5 Department of Vascular Biology, Children's Hospital and Harvard Medical School, Enders Building, 10th Floor, 300 Longwood Avenue, Boston, MA, USA

## Abstract

**Introduction:**

The dynamics of breast cancer recurrence and death, indicating a bimodal hazard rate pattern, has been confirmed in various databases. A few explanations have been suggested to help interpret this finding, assuming that each peak is generated by clustering of similar recurrences and different peaks result from distinct categories of recurrence.

**Methods:**

The recurrence dynamics was analysed in a series of 1526 patients undergoing conservative surgery at the National Cancer Institute of Milan, Italy, for whom the site of first recurrence was recorded. The study was focused on the first clinically relevant event occurring during the follow up (ie, local recurrence, distant metastasis, contralateral breast cancer, second primary tumour), the dynamics of which was studied by estimating the specific hazard rate.

**Results:**

The hazard rate for any recurrence (including both local and distant disease relapses) displayed a bimodal pattern with a first surge peaking at about 24 months and a second peak at almost 60 months. The same pattern was observed when the whole recurrence risk was split into the risk of local recurrence and the risk of distant metastasis. However, the hazard rate curves for both contralateral breast tumours and second primary tumours revealed a uniform course at an almost constant level. When patients with distant metastases were grouped by site of recurrence (soft tissue, bone, lung or liver or central nervous system), the corresponding hazard rate curves displayed the typical bimodal pattern with a first peak at about 24 months and a later peak at about 60 months.

**Conclusions:**

The bimodal dynamics for early stage breast cancer recurrence is again confirmed, providing support to the proposed tumour-dormancy-based model. The recurrence dynamics does not depend on the site of metastasis indicating that the timing of recurrences is generated by factors influencing the metastatic development regardless of the seeded organ. This finding supports the view that the disease course after surgical removal of the primary tumour follows a common pathway with well-defined steps and that the recurrence risk pattern results from inherent features of the metastasis development process, which are apparently attributable to tumour cells.

## Introduction

The dynamics of disease recurrence has been investigated previously in a series of 1173 patients undergoing mastectomy as single initial treatment for early stage breast cancer at the National Cancer Institute of Milan, Italy [1-3]. The hazard rate for recurrence indicated a bimodal relapse pattern with an early, rather sharp, dominant peak at about two years and a second broader peak at about five years with a decay that extended to at least 15 years. The finding has been confirmed in other databases for both recurrence [4-8] and mortality [9-13], and was even evident when patients received adjuvant chemotherapy [8]. It was not predicted by any prevailing theory of breast cancer evolution that was considered to have originated from unrestrained continuous cellular growth. The multipeak hazard rate for recurrence implied heterogeneity of treatment failure.

To explain the bimodal pattern, a simple metastasis development model was proposed [14,15] consisting of three distinct phases: a single malignant cell, an avascular lesion and a vascularised growth. Tumour dormancy was assumed to be possible at the single cell level and also at the point where, for further growth, angiogenesis is necessary [14]. The model was able to simulate [15] the second peak as a steady stochastic progression from one phase to the next. In order to simulate the first peak, however, it was postulated that for some subsets of patients, transitions from one state to the next were stimulated at or about the time of surgery. The conclusions were that the dominant mode of relapse in early stage breast cancer was the result of events terminating dormancy phases at the time of surgery.

Other explanations for the bimodal hazard rate pattern have also been suggested. Attempts to evaluate the role of local failure in distant failure and survival led to the hypothesis that the second recurrence surge originated by distant metastases started by a prior local failure, involving delicate issues in the formalisation and interpretation of the analyses [5]. Researchers analysing breast cancer mortality have suggested that bimodal patterns could result from the heterogeneous "malignant potential" of breast cancer [12]. According to this latter hypothesis, the two mortality peaks are attributable to different types of failure caused either by early and late tumour relapse or by local tumour recurrence and distant metastasis. Underlying these explanations is the concept that the hazard rate pattern should be explained by a juxtaposing of populations with different, yet uniform, disease development. They suggest that each peak is generated by clustering similar recurrences while different peaks result from distinct categories of recurrence. Instead of that explanation, the proposed tumour-dormancy-based model is essentially saltatory and suggests that the occurrence of different peaks is generated by the intrinsic general process of the metastatic development, although the underlying mechanism still remains unclear.

In the present study we analysed the recurrence dynamics in a further series of patients undergoing conservative surgery at the National Cancer Institute of Milan, for whom the site of first recurrence was recorded. The study provides evidence that different categories of metastases display the same bimodal hazard rate pattern. This finding supports the view that the disease course after surgical removal of a primary tumour follows a common pathway with well-defined steps and that the recurrence risk pattern results from inherent features of the metastasis development process, which are apparently attributable to tumour cells.

## Materials and methods

### Patients

The preliminary results of the Milan trial comparing quadrantectomy plus radiotherapy (QUART) to mastectomy were obtained in 1980, and since then routine practice at the National Cancer Institute of Milan has been conservative treatment of early breast cancer, following informed consent of the patient. Patients undergoing the clinical treatment with QUART, who were not included in a randomised clinical trial and who met the same criteria implemented for trial cases [16], were included in the study (out-trial patients). Briefly, out-trial patients with unilateral primary breast cancer up to 3.5 cm in diameter, clinically uninvolved axillary lymph nodes and no other evidence of tumour spread received QUART. Quadrantectomy was performed by removing the primary tumour and a 2 to 3 cm margin of normal mammary tissue. Axillary lymph nodes were completely excised. Radiotherapy to the ipsilateral breast (50 Gy with high energy plus 10 Gy as a boost with orthovoltage) was started within one month of surgery. Women with histologically positive axillary nodes were allocated to receive systemic adjuvant treatment.

Patients were followed up quarterly for the first five years and then twice a year. Chest x-ray was performed every six months for the first five years and then every year. Bone and liver scans and mammographies were performed every year. If any symptoms or signs suggestive of a potential recurrence were detected or reported by the patients, focused investigations were carried out. If recurrence was documented, a complete restaging was obtained. All baseline data, treatment features and relevant clinical events were collected in standard format and stored in a clinical database. Data about a few currently assessed biological markers (ie, oestrogen receptor (ER), progesterone receptor (PR) and human epidermal growth receptor 2 (HER2)) were not systematically recorded and were not considered in the analysis. Also, information about familial risk factors was not available.

This study focused on the first clinically relevant event occurring during the follow-up period, that is local recurrence, distant metastasis, contralateral breast cancer and second primary tumour. Local recurrence was defined as any new breast cancer appearance in the breast already operated on only. Distant metastasis was defined as any breast cancer manifestation(s) in areas other than that of local recurrence with the exception of the contralateral breast, where it was defined as contralateral breast cancer. Primary malignant tumours in other organs were defined as second primaries. Distant metastases were categorised as bone, viscera and soft tissue recurrences according to previously defined criteria [17]. In the case of synchronous visceral and non-visceral localisations the recurrence was recorded as multiple visceral. Soft tissue metastases also included the supra-clavicular lymph node recurrences.

### Statistical analysis

The recurrence dynamics was studied using the life-table method to estimate the hazard rate for recurrence, that is, the conditional probability of manifesting recurrence in a time interval, given that the patient is clinically free of any recurrence at the beginning of the interval. We applied a discretization of the time axis in a variety of units. Each calculated value represents the measure of the hazard rate for recurrence within the considered time unit. Although the hazard rate estimates display some instability due to random variation, a Kernel-like smoothing procedure [18] was adopted to aid the interpretation of the underlying pattern, and the smoothed curves were graphically represented. Different time intervals were utilised in a preliminary smoothing analysis that showed three-month intervals were a good compromise between smoothing data and displaying the underlying structure. Therefore, all hazard rate levels were measured as 'events/patients at risk per three-month interval'.

In addition to the kernel smoothing approach with discrete hazards, a formal flexible regression modelling strategy was adopted as proposed by Boracchi and colleagues [19]. Because of the exploratory nature of the present study, B-spline transformations over time were used [20] instead of the truncated power spline notation approach, to allow for smoothed multimodal hazard patterns over the entire follow-up length. To account for different behaviours according to the specific event of interest, the statistical models jointly analysed all the events, allowing for interactions between the time bases and event indicators, considering B-spline bases with degrees of freedom (df) ranging from 4 to 10. Therefore, the evidence of different patterns according to different events was informally assessed by selecting the best models according to the Akaike Information Criterion (AIC) (Tables 1 and 2).

**Table 1:** Hazard rate model AIC values for the analysis of distant recurrences, local recurrences, contralateral breast cancer and other primary tumours

4	234.9	212.2
**5**	224.8	**208.4**

6	225.2	221.2

7	227.4	218.8

8	228.1	224.5

9	230.2	231.2

10	230.9	235.6

The table reports Akaike Information Criterion (AIC) values according to the different models. The minimum value is reported in bold with the corresponding degrees of freedom indicating the informally selected model.

**Table 2:** Hazard rate model AIC values for the analysis of distant recurrences in different sites

Spline degrees of freedom	AIC (models without interaction)	AIC (Models with interaction)
5	110.1	117.9

**6**	**106.4**	112.3

7	106.8	115.4

8	106.9	117.5

9	111.5	120.5

10	113.0	124.1

The table reports Akaike Information Criterion (AIC) values according to the different models. The minimum value is reported in bold with the corresponding degrees of freedom indicating the informally selected model.

## Results

A total of 1526 patients who received QUART during the 10 years between 1974 and 1984 and were not included in any other clinical trial are incorporated in this analysis. The main patient characteristics are summarised in Table 3. Patients were generally young (41% less than 45 years) premenopausal (60%) women with small tumours (90% T1), who underwent a uniform treatment with standard QUART delivered by the same clinical team. Adjuvant cyclophosphamide, methotrexate plus fluorouracil therapy was administered to most (70%) node-positive cases.

**Table 3:** Main patient characteristics

Total number	1526
Age (years)	
≤ 45	625
46 to 55	468
56 to 65	283
> 65	150
Menopausal status	
Pre	922
Post	594
Unknown	10
Tumour size	
≤ 1 cm	587
1.1 to 2 cm	779
2.1 to 3 cm	143
> 3 cm	17
Nodal status	
N-	964
1 to 3 N+	416
>3 N+	146
Adjuvant therapy for N+ patients	
None	45
CMF	394
Tamoxifen	30
Other	3
Unknown	90

N- = axillary node negative; N+ = axillary node positive; CMF = cyclophosphamide, methotrexate plus fluorouracil therapy.

The first event was local recurrence in 119 patients and distant metastasis in 280 patients, and in 73 cases a contralateral breast cancer was first recorded and further 39 patients developed a second primary as first event.

**Figure 1:**
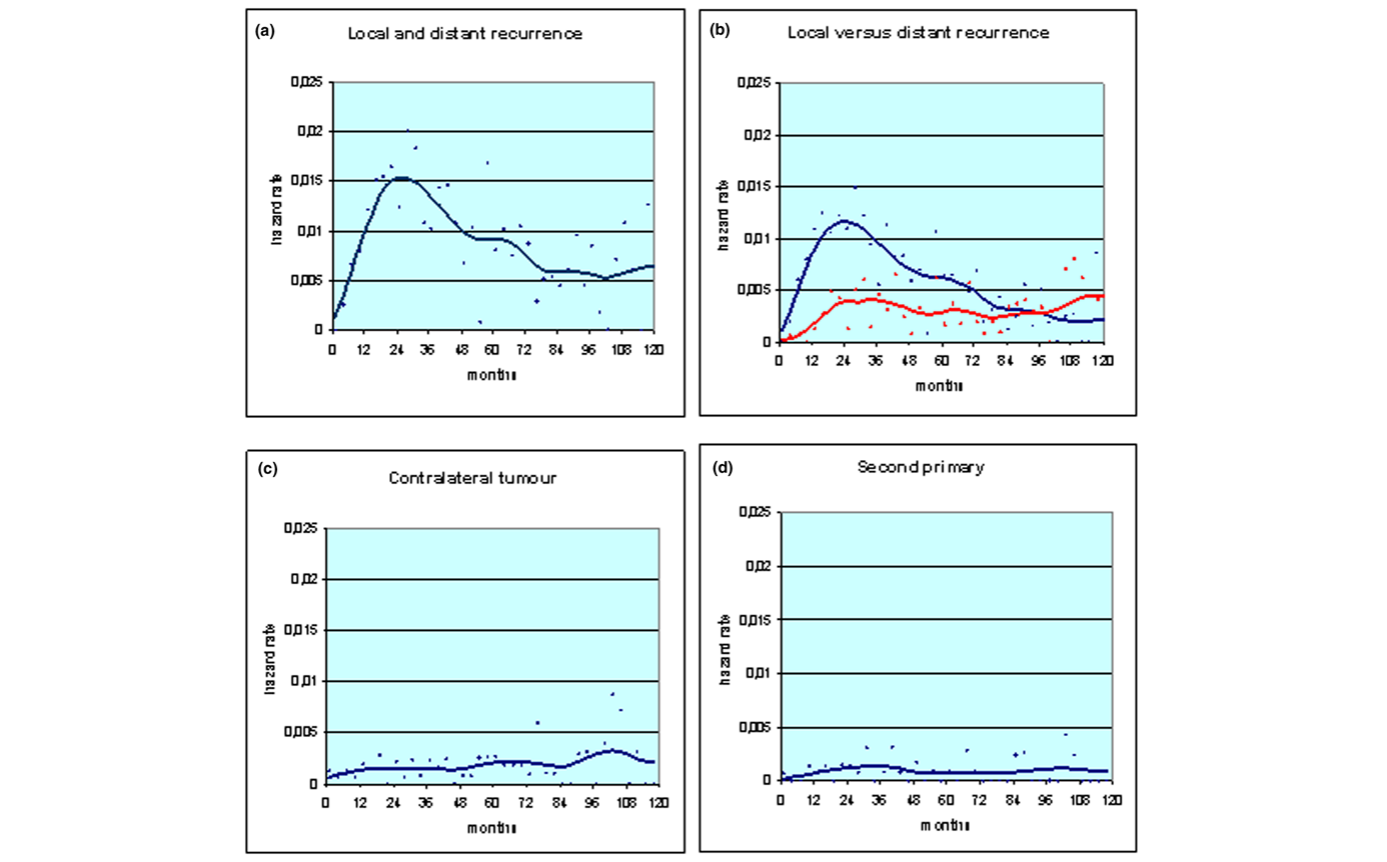
**Hazard rate estimates for selected events in 1526 patients undergoing conservative surgery**. Each point represents the measure of the hazard rate of the given event within a three-month interval. The smoothed curve was obtained by a Kernel-like smoothing procedure. (a) Hazard rate for any recurrence (including both local and distant disease relapses). (b) The hazard rate for recurrence is split into its components: local recurrence (red line) and distant metastasis (blue line). (c) Hazard rate for contralateral breast cancer. (d) Hazard rate for second primary cancer.

The hazard rate for any recurrence (including both local and distant disease relapses) displayed a bimodal pattern with a first surge peaking at about 24 months (estimated risk value = 0.016) and a second peak at almost 60 months (estimated risk value = 0.009) (Figure 1a). The same pattern was observed when the whole recurrence risk was split into its components: the risk of local recurrence and the risk of distant metastasis (Figure 1b). The hazard rate curves for both contralateral breast tumours and second primary tumours did not show major peaks but revealed an almost uniform course at a quite constant level of about 0.002 for contralateral breast tumours and about 0.001 for second primary tumours (Figures 1c and 1d).

**Figure 2:**
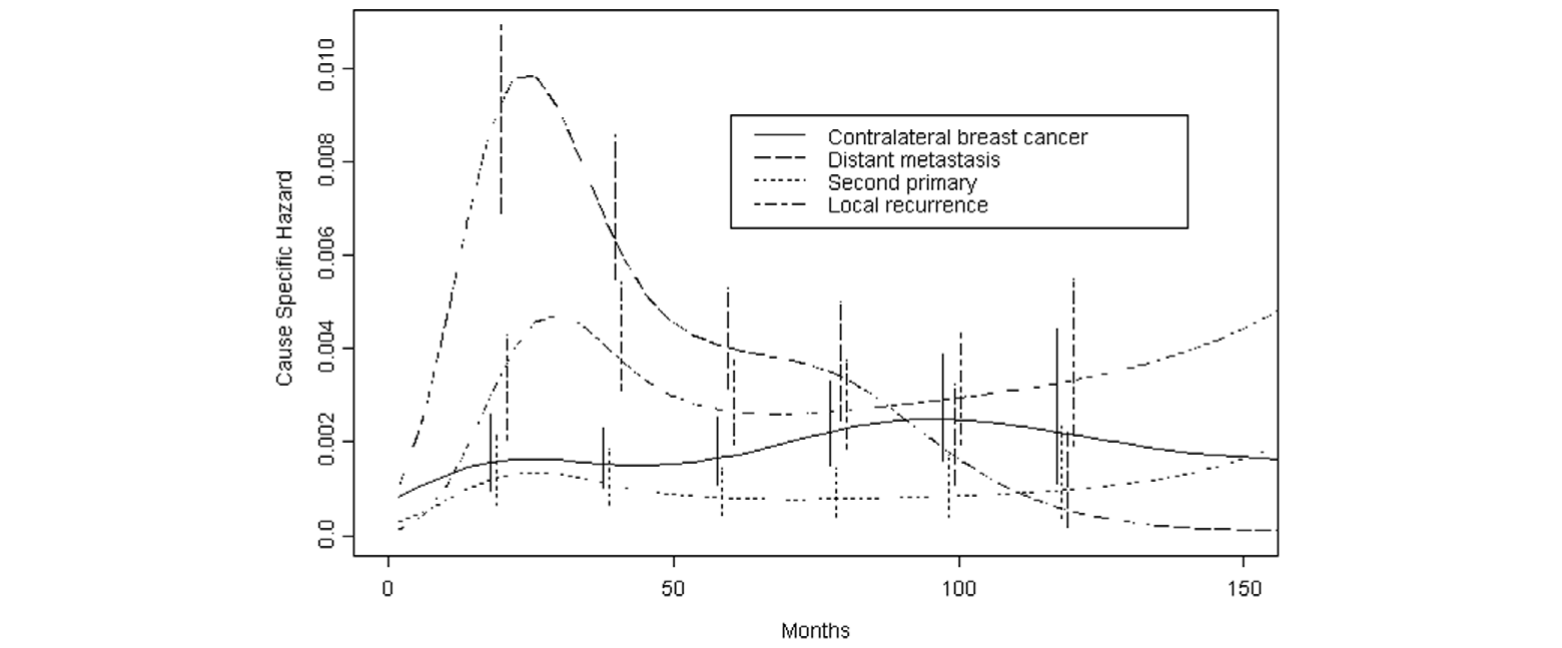
**Hazard rate for selected events in 1526 patients undergoing conservative surgery**. The same events as in Figure 1 were analysed by a formal flexible regression modelling strategy considering B-spline bases with degrees of freedom ranging from 4 to 10 and selecting the best models according to the Akaike Information Criterion. Vertical lines represent point-wise confidence interval for the model estimated hazards, according to standard asymptotic theory.

The pattern of the hazard function for the analysed events was confirmed by the flexible regression spline models. The selected model, according to the AIC, had 5 df on time including the interaction with the event type indicator, thus supporting the evidence of a different hazard shape behaviour according to the different events. The estimated cause specific hazard curves from the selected interaction models are reported in Figure 2. The bimodal behaviour of distant metastasis is also evident from such an analysis, as well as the uniform tendency of contralateral breast cancers and second primaries. With regard to local recurrences, the analysis did not yield any evidence of a second peak. However, this fact does not imply the absence of such a pattern because of the parametric nature of the regression modelling approach, focused on major effects rather than local behaviours according to the available sample information.

**Figure 3:**
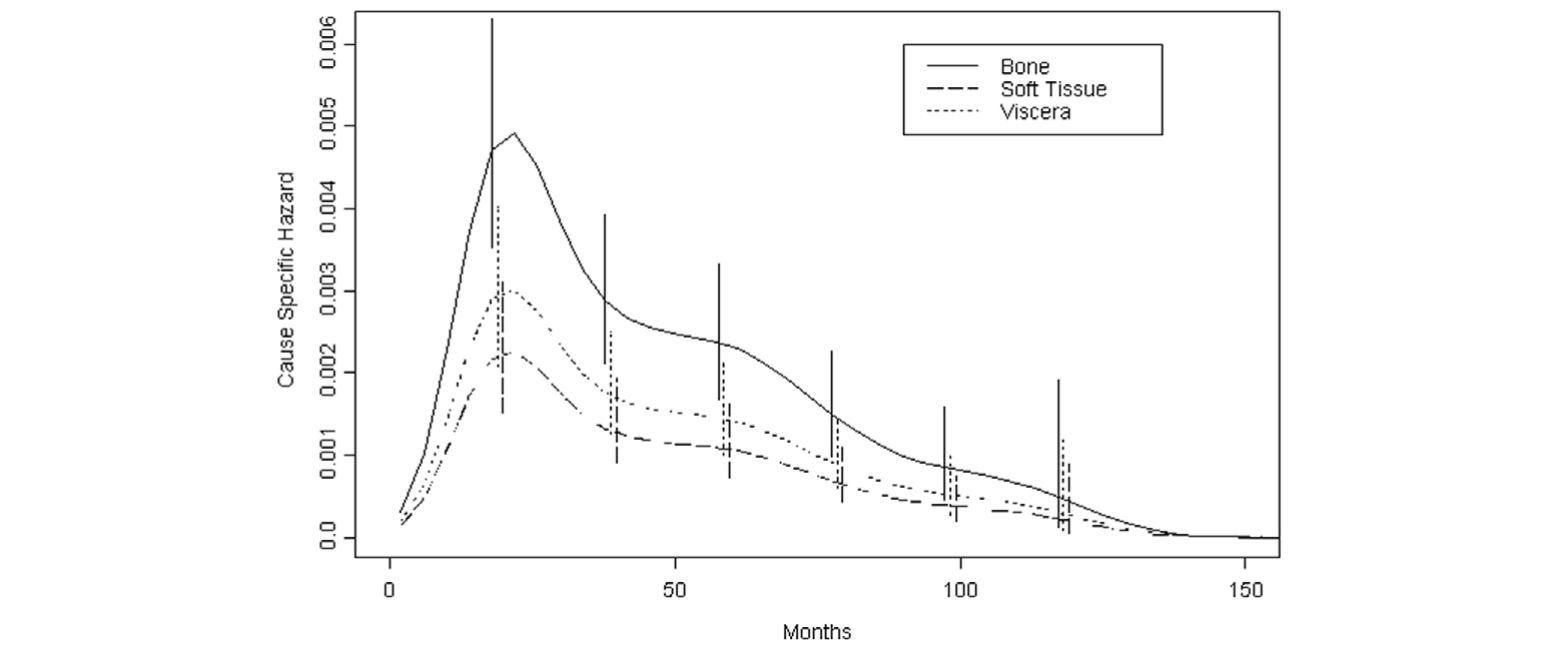
**Hazard rate for distant metastasis in different sites**. Distant metastases were categorised as bone, viscera and soft tissue recurrences. Soft tissue metastases also included the supra-clavicular lymph node recurrences. Because of the limited number of events to a single visceral site, recurrences to lung, liver or CNS were merged to obtain a more suitable collection of cases, representative of the visceral recurrence. Vertical lines represent point-wise confidence intervals for the model estimated hazards, according to standard asymptotic theory.

Among patients with a first recurrence at a distant site, 98 women showed bone metastasis only, 45 cases had clinically evident foci in soft tissue(s) and in a further 135 patients the disease reappeared in visceral sites, either as single organ involvement or as multiple recurrence in association with other visceral, soft tissue or bone localisations. According to the study aims, the assessment of recurrence dynamics should have been focused on each single site. However, because of the limited number of events to a single visceral site, recurrences to lung, liver or CNS were merged to obtain a more suitable collection of 60 cases, representative of the visceral recurrence. Therefore, three subsets of distantly recurring patients (to soft tissue, bone, lung or liver or CNS) were analysed. The selected model, according to the AIC, had 6 df on time without including the interaction with the recurrence site indicator and did not support any evidence of a different hazard shape behaviour according to the different events. The corresponding hazard rate curves displayed the typical bimodal pattern (Figure 3). In particular, the position of the first peak on the time axis is at about 24 months while the later peak emerges at about 60 months for all recurrence sites.

## Discussion

The results of this study confirm previous findings and include further details of the metastatic process, providing additional support to the proposed tumour-dormancy-based model. In particular, the results show that peaks are not determined by similar clustering of given categories of metastasis and suggest that the different timing of recurrences is generated by factors influencing the metastatic development regardless of the seeded organ. Regrettably, analysed data were lacking information about some important biomarkers such as ER, PR and HER2 that could have provided more information about mechanisms underlying the bimodal kinetics.

The bimodal recurrence risk pattern is once again emerging from the clinical data of this new series of patients as a current feature of the recurrence dynamics. The peak position on the time axis is unchanged in comparison to the findings from the previously analysed series of patients [3]. In the present analysis we found lower peaks estimating the three-month recurrence risk level (0.016 versus 0.033 for the first peak and 0.009 versus 0.014 for the second peak) than in the previous study. This finding is well explained by the different characteristics of the two studied populations. Indeed, this analysis included fewer patients with a poor prognosis (10% tumour size of 2 cm or more, 37% N+, no adjuvant chemotherapy for 30% N+ patients) than the previous one (60% tumour size 2 cm or more, 49% N+, no adjuvant chemotherapy for any N+ patient) [3], accounting for the observed differences. When comparing the present series to the T1 subset of the previous series, even peak height differences almost do not exist (data not shown).

As previously observed [2], both contralateral breast cancers and second primaries display a quite constant hazard rate pattern, confirming that the occurrence of contralateral breast cancer should be considered a 'memory-less' stochastic event unrelated to the primary tumour [21,22] or tumours developing in other organs. This concept is further strengthened by the estimated annual risk level, which is greater for contralateral breast tumours than for other second primaries, as it may be expected in these patients who should be considered at higher-than-average risk for breast cancer (Figures 1c, d and 2).

The hazard rate for local recurrence presents a bimodal curve analogous to the curve of distant metastasis. The two curves cross at about eight years, when the risk of local recurrence ceases to decrease, while the hazard rate for distant metastasis goes on to regularly drop (Figures 1b and 1c). In previously analysed patients who underwent mastectomy [2], the risk of local recurrence showed a definite decreasing pattern after the second peak and promptly reached an almost null level. It should be taken into account, however, that women undergoing QUART maintain a significant portion of their mammary gland while in patients having mastectomy the breast area is totally cleared of breast tissue. Therefore, patients who undergo QUART may develop a further breast primary (sometimes from ductal carcinoma in situ) in the residual parenchyma while patients who undergo a mastectomy do not. According to this hypothesis, which was devised since the publication of the early reports on breast cancer conservative surgery [16,23] and repeatedly discussed in further reports [24-27], the hazard rate for local recurrence is the superimposition of the curve resulting from the true local recurrences and the straight line paralleling the time axis resulting from the memory-less stochastic appearance of a further ipsilateral breast cancer. The resulting hazard rate curve, therefore, is expected to show a right-sided tail at an almost constant level, as in fact occurs (Figures 1b and 2). Distinguishing between true recurrence and second ipsilateral primary is clinically relevant and has been widely pursued [26-29], although without firm results until now.

The analysis of the hazard rate for distant metastases to different organs consistently suggests that the recurrence dynamics does not depend on the site of metastasis. This occurrence supports the concept that the recurrence risk pattern results from inherent features of the metastasis development process, which are apparently attributable to tumour cells, although the local micro-environmental host conditions should be permissive for further metastasis growth. This result has been partially anticipated [2] by the bimodal risk pattern for local recurrence, which may be viewed as a type of soft tissue metastasis in patients undergoing mastectomy and lacking residual breast parenchyma. However, no firm conclusion had been reached given that local recurrences may develop even from tumour cell deposits subsequent to incomplete surgical clearing, therefore not being representative of the metastatic process. The results presented here remove any doubt about this issue and suggest that some traits of the metastasis development process are similar in all seeded organs.

The present findings provide new elements for a reassessment of previously proposed explanations of the bimodal hazard rate pattern, all assuming a uniform development of the microscopic disease. The explanation suggested by Fortin and colleagues [5] fails to elucidate both the present and previous [2] findings: their opinion that the "second peak can be explained only by a second event, namely local failure" are not valid for findings obtained from our analyses that are focused on first events. Beyond this methodological drawback, however, we wish to emphasise that the proposed explanation implies that an observed peak should be related to patients dynamically clustered (eg, patients displaying local recurrence). A similar conceptual criticism can be addressed to the work by Yakovlev and colleagues [12], who found a two-component structure of the hazard function in breast cancer survival and suggested possible explanations based on the heterogeneity of "malignant potential" remaining in treated tumours. The suggested explanations, that patients with different types of failure (more vs less rapidly evolving disease) or different site of failure (local vs distant) produce different peaks, assume that each peak is generated by the clustering of cases with similar features, although different peaks result from distinct categories of patients.

The results of the present study argue against these views and support the concept that different peaks are related to the intrinsic general pathway of the metastasis development, not to distinct categories of recurrence. Indeed, our results provide evidence that the recurrence dynamics in different metastatic sites is similar to the recurrence dynamics found in different patient subsets [2,3,8], suggesting that the disease course after surgical removal of a primary tumour apparently follows a basic common pathway with well-defined steps. The proposed tumour-dormancy-based model recognises such steps as metastatic dormant states at the single cell level and avascular micrometastasis level [3,8,14], and relates the hazard rate pattern for recurrence to the non-linear disease development. Within the common rhythm of the recurrence dynamics, the risk levels at a certain time are influenced by tumour and host traits [3,14], suggesting that the pace of the common pathway is governed by a specific mixture of factors.

The delayed appearance of metastases has driven several explanations, a few of which supported by the results of sophisticated molecular techniques such as whole-genome analysis or gene expression profiling [30,31]. Ductal carcinoma in situ lesions and even disseminated tumour cells seeding distant sites would need extra time to cumulate additional genetic progression and develop into invasive cancers. At present, however, it remains unclear how such mechanisms may account for the observed recurrence dynamics with its cadences. Future studies are needed to find connections between these explanations and the tumour dormancy hypothesis, which at present seems to fit the clinical findings adequately.

## Conclusion

In the present study we analysed the recurrence dynamics in a series of patients undergoing conservative surgery at the National Cancer Institute of Milan. The recurrence dynamics was studied with the life-table method to estimate the hazard rate for recurrence, that is, the conditional probability of manifesting recurrence in a time interval, given that the patient is clinically free of any recurrence at the beginning of the interval. Moreover, the analysis was focused on the first clinically relevant event occurring during the follow up, that is, local recurrence, distant metastasis, contralateral breast cancer and second primary.

The bimodal recurrence risk pattern emerged from the clinical data of this new series of patients as a current feature of the recurrence dynamics. The peak position on the time axis was unchanged in comparison to the findings from the previously analysed series of patients, with an early peak at about two years and a second broader peak at about five years with a decay afterwards.

Three subsets of distantly recurring patients (to soft tissue, bone and viscera) were analysed and the analysis did not support any evidence of a different hazard shape behaviour according to the different events. The corresponding hazard rate curves displayed the typical bimodal pattern and, in particular, the position of the first peak on the time axis was at about 24 months, while the later peak emerged at about 60 months for all recurrence sites.

The study provides evidence that different categories of metastases display the same bimodal hazard rate pattern. Therefore, the concept that different peaks result from distinct categories of patients such as patients with different types of failure (eg, more vs less rapidly evolving disease) or different site of failure (eg, local vs distant or viscera vs bone) fails to be supported. Rather, the study maintains the view that breast cancer course after surgical removal of a primary tumour follows a common pathway with well-defined steps and that the recurrence risk pattern results from inherent features of the metastasis development process, which are apparently attributable to tumour cells, although the local host micro-environmental conditions should be permissive for further metastasis growth.

## Abbreviations

AIC: Akaike Information Criterion; df: degrees of freedom; ER: oestrogen receptor; HER2: human epidermal growth factor receptor 2; N-: axillary node negative; N+: axillary node positive; PR: progesterone receptor; QUART: quadrantectomy plus radiotherapy.

## Competing interests

The authors declare that they have no competing interests.

## Authors' contributions

RD conceived the study, and led the analysis, interpretation of results and the drafting of the manuscript. EB and PB performed the statistical analysis and were involved in the drafting of the manuscript. MG was involved in the acquisition of data. MWR was involved in drafting and critically revising the manuscript.
